# Evidence-based construction and validation of an agent-based model of community integrated care for older adults living with dementia

**DOI:** 10.3389/fpubh.2026.1913457

**Published:** 2026-09-23

**Authors:** Chenli Zhu, Jingxuan Gao, Haijin Li, Hui Li, Bingjie Chang, Shuang Shao, Juan Du

**Affiliations:** School of General Practice and Continuing Education, Capital Medical University, Beijing, China

**Keywords:** agent-based modeling, community health services, dementia care, health services research, integrated care, primary care

## Abstract

**Objective:**

Community dementia care involves interacting patients, caregivers, and formal care providers whose long-term dynamics are difficult to characterize with single-component empirical studies. We constructed and assessed a baseline agent-based model (ABM) to establish a transparent, inspectable reference scenario against which future computational experiments on organizational configurations can be evaluated.

**Methods:**

A fully rule-based ABM of community dementia care was developed using prior empirical studies, published literature, policy documents, and structured expert consultation. Each rule and parameter was documented with its evidence source and population provenance. The formal baseline used 1,000 synthetic care units, 100 independent replications, a 60-month horizon, and all configuration switches at baseline values. Behavioral and psychological symptoms of dementia (BPSD) prevalence at initialization was aligned with a prespecified Chinese meta-analytic anchor.

**Results:**

BPSD prevalence increased from 75.6% at initialization to 96.4% at month 60 (Monte Carlo 95% CI, 96.3–96.5%). Mean caregiver burden rose from 0.505 to 0.782. By month 60, a mean of 161 care units were in institutional care. Scale and replication diagnostics confirmed numerical adequacy; local sensitivity analysis showed limited changes in primary outcomes across the tested range.

**Conclusion:**

Under the documented formal baseline rules, the model generates conditional trajectories of disease progression, caregiver burden, closed-loop referral completion, and institutional-care membership. The resulting transparent baseline provides a comparison condition for future, explicitly scoped computational experiments on organizational configurations.

## 1 Introduction

Dementia involves progressive cognitive decline, loss of activities of daily living, and behavioral and psychological symptoms, entailing sustained care across medical, rehabilitative, and social-support domains (1). The global population living with dementia is projected to grow from approximately 57 million in 2019 to around 153 million by 2050 (2). Integrated care is widely advocated as a response to fragmentation, emphasizing coordination across clinical, organizational, and financing dimensions (3, 4). At the community level, however, implementation is constrained by limited provider time, the low priority of dementia in general practice, collaboration dependent on personal relationships rather than institutional arrangements, and payment systems that do not support coordination (5).

In China, barriers to community dementia care are shaped by the organization of primary care, specialist services, family care, and social support. Dementia diagnosis and management at the primary-care level remain insufficient, with barriers spanning providers’ capability, opportunity, and motivation (6); patients may bypass primary care and seek care directly from general hospitals (7); and demographic change and the growth of empty-nest households place pressure on family care (8). Although the policy agenda for medical–social care integration has been established, implementation at community level remains uneven (9, 10).

Intervention studies and quasi-experimental designs are essential for estimating the effects of specific care arrangements (11), but their application is difficult when the object of inquiry is a multi-actor system with interacting components and outcomes that unfold over long horizons. Agent-based modeling (ABM) represents heterogeneous agents with explicit attributes and behavioral rules, generates system-level patterns from local interactions, and permits formal computational experiments that vary system configurations while holding other conditions constant (12–14). In community dementia care, ABM can represent the feedback among patients, caregivers, and service providers, and can later explore alternative organizational arrangements through scenario experiments (15). Model credibility depends on transparent structural documentation, traceable evidence-to-parameter mapping, and interpretation proportionate to the evaluation evidence (16).

Existing dementia simulations have concentrated on disease natural history, resource projection, or cost-utility evaluation using state-transition approaches (17, 18). Fewer models explicitly represent the interactions among caregivers, primary-care providers, specialists, and organizational arrangements, and many rely on parameters drawn from systems outside the target setting (19, 20). Before an ABM is used for policy-oriented scenario analysis in China’s primary-care context, its structure, evidence-to-parameter mapping, initialization, and baseline system behavior require transparent description and assessment.

Accordingly, this study reports the construction and baseline assessment of a fully rule-based ABM of community dementia care. The model is designed as a computational experiment platform: a transparent, inspectable representation of the care system against which the consequences of alternative organizational configurations can later be formally examined. The present analysis describes the model structure and evidence-to-parameter mapping, and evaluates the baseline scenario through initialization calibration, convergence diagnostics, local sensitivity analysis, and pattern-oriented assessment. The study examines whether the specified rule set generates plausible system-level patterns under the modeled baseline conditions.

## 2 Materials and methods

### 2.1 Model purpose and design overview

The purpose of the Dementia Care ABM was to represent community dementia care as a system of interacting patients, caregivers, providers, and institutions, and to generate a transparent, inspectable baseline configuration against which future scenario analyses could be evaluated. The present analysis focused on model construction, initialization calibration, convergence diagnostics, and pattern-oriented assessment of the baseline scenario.

All decision-making processes were implemented as pre-specified rules, thresholds, or probabilistic functions parameterized through log-odds weights; no large language model generated decisions during simulation. This design was selected to make the correspondence between evidence, behavioral assumptions, and model outputs inspectable and reproducible. Given the same code version, configuration, synthetic input cohort, and random-seed set, the simulation generated the same outputs.

Caregiver behavior was represented as bounded decision-making rather than utility maximization. Help-seeking and service-use intentions were generated from explicit contributions of need, prior experience, trust, economic circumstances, and caregiving burden. Model inputs were assembled from four prior empirical studies and one structured expert consultation conducted by the research group (Q1–Q5; Table 1), published clinical and health-services literature, and policy documents. Each parameter and rule was recorded in an evidence registry with its source, population provenance, evidence-strength assessment, and linked model mechanism (Supplementary methods S1). The complete ODD+D description (21, 22), parameter evidence grading, and rule specifications are provided in the Supplementary methods S2.

**Table 1:** Prior studies supporting the model and their primary mapping onto model components.

ID	Study	Design	Sample	Primary mapping target
Q1	Care experience of general practitioners and family caregivers	Qualitative interviews using the RMIC framework	10 general practitioners and 11 family caregivers	Baseline behavioral logic and rule direction
Q2	Survey of community care-service capacity	Cross-sectional quantitative survey	78 community health centers, 1,007 primary-care physicians, and 954 community nurses	Institutional capacity and provider-related parameters
Q3	Survey of family-caregiver service utilization	Cross-sectional questionnaire survey	202 family caregivers	Caregiver characteristics, service-use patterns, and selected quantitative inputs
Q4	Systematic review of facilitators and barriers to integrated care	Qualitative systematic review	24 international papers	Generic contextual mechanisms
Q5	Structured expert consultation	Structured qualitative consultation	17 stakeholders across six categories	Contextual plausibility and operational specification of rules

The detailed evidence-to-parameter mapping and evidence-strength assessment are provided in the Supplementary methods S1.

### 2.2 Entities, state variables, and scales

The care unit was the primary unit of analysis and comprised a person living with dementia and a family caregiver. Patient state variables included cognitive impairment stage (Clinical Dementia Rating [CDR] 0.5, 1.0, 2.0, 3.0), activities of daily living (ADL, 0–100), BPSD status (presence, symptom count, severity), hospitalization count, and institutionalization status. Caregiver state variables included demographic and socioeconomic characteristics, caregiving intensity, and a continuous caregiver burden score (0–1) updated monthly based on care intensity, disease progression, and service-use outcomes; its initialization and update rules are documented in the Supplementary material. Patients were represented through state-transition processes rather than as autonomous decision-making agents.

The model represented five types of agents: family caregivers, community general practitioners (GPs), hospital specialists, care coordinators, and residential care institutions. Caregivers were heterogeneous in demographic, socioeconomic, and caregiving characteristics and acted through the rule engine. GP heterogeneity was represented across three competence dimensions: assessment, medication management, and BPSD management. When the learning-organization configuration was enabled, GP competence increased at institution-stratified rates. Care coordinators were available only when the corresponding configuration was enabled and had limited contextual heterogeneity: a fixed, deterministically sampled fraction of care units was assigned a social-worker-type coordinator whose support competence met a specified minimum; other coordinator support depended on institutional competence. Individual differences in coordinator background, experience, workload, and practice style were not represented. Individual-level heterogeneity among hospital specialists was likewise intentionally outside the model boundary; specialists functioned as diagnosis, referral-receipt, feedback, and return-to-community nodes.

The temporal scale was monthly, with a 60-month horizon. The spatial scale was the community care system. The model contained no spatial grid; care-unit proximity to services was represented through service visibility and economic accessibility parameters rather than geographic distance.

### 2.3 Process overview and scheduling

The model advanced in monthly time steps for 60 months. Each epoch followed a fixed seven-phase schedule (Phases 0–6; Figure 1): (0) time setting and monthly signal reset; (1) natural disease progression; (2) proactive processes (disabled in baseline); (3) caregiver rule-based decisions; (4) formal service responses, including GP assessment, referral, and specialist handling; (5) subsystem updates and institutionalization review; and (6) outcome collection. This ordering specified the causal sequence through which changes in patient state and caregiver circumstances could trigger assessment, referral, service responses, and subsequent feedback.

**Figure 1:**
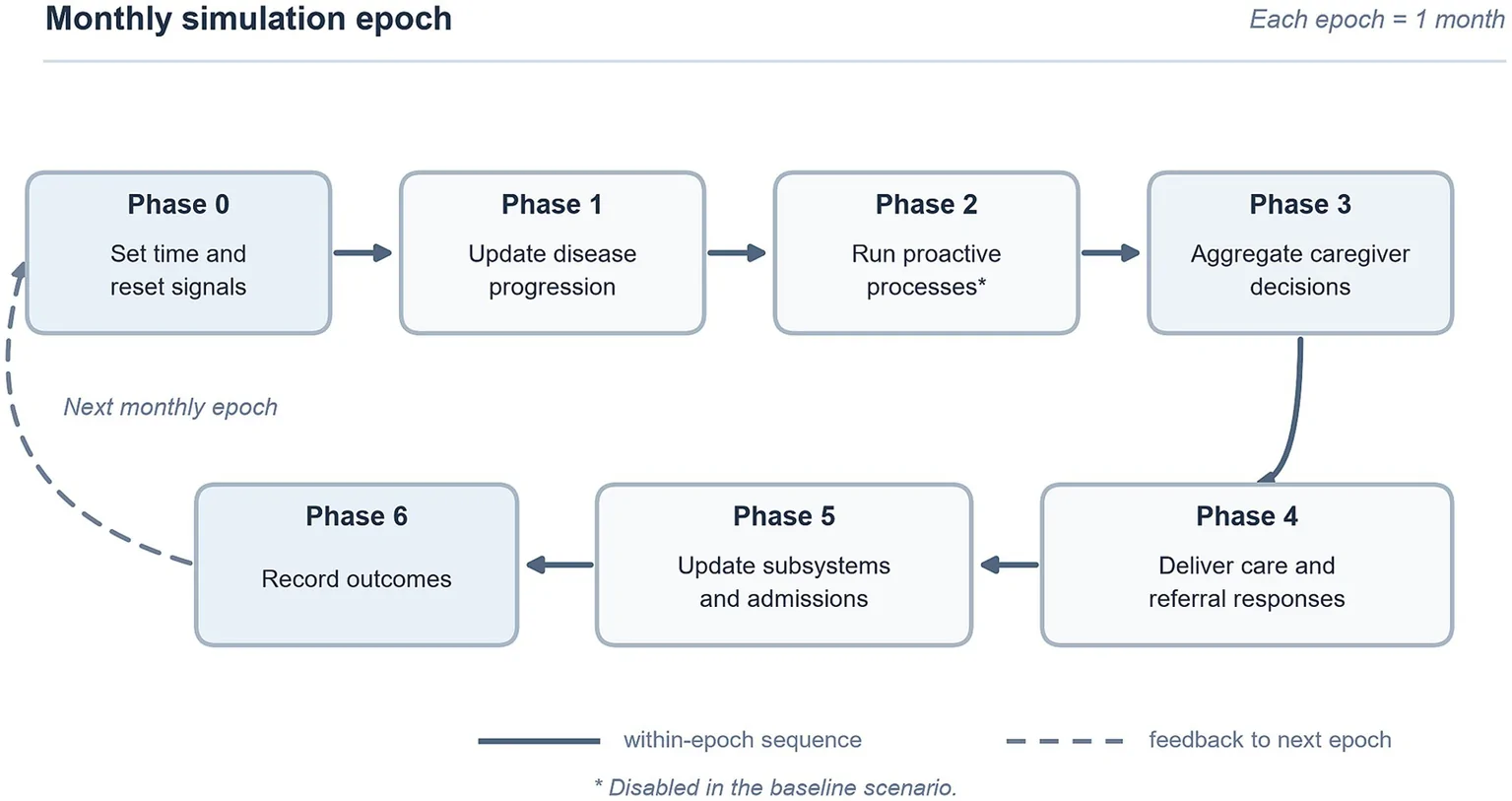
Seven-phase monthly simulation schedule. Each epoch represents one simulated month. Phase 0 sets subsystem time and resets monthly signals; Phase 1 updates natural disease progression; Phase 2 executes proactive processes, which are disabled in the formal baseline; Phase 3 aggregates caregiver rule-based decisions; Phase 4 applies GP, referral, specialist, and institutional rule-agent responses; Phase 5 updates subsystems and confirms institutional admissions; and Phase 6 records outcomes. Solid arrows indicate the within-epoch sequence, whereas the dashed arrow indicates feedback into the next monthly epoch. After institutional admission, a care unit no longer contributes to community-service, new-referral, help-seeking, or caregiver-burden outcomes.

At initialization (month 0), patient and caregiver states were loaded before the first natural-history update. Accordingly, month 0 for the CDR stage distribution, BPSD prevalence, and mean ADL denotes the initialized cohort state, and months 1–60 denote states recorded after the corresponding monthly update. Caregiver burden was recorded after each monthly caregiver-decision step and was therefore available from month 1 onward. These time-indexing conventions were used for the trajectories displayed in Figure 2.

**Figure 2:**
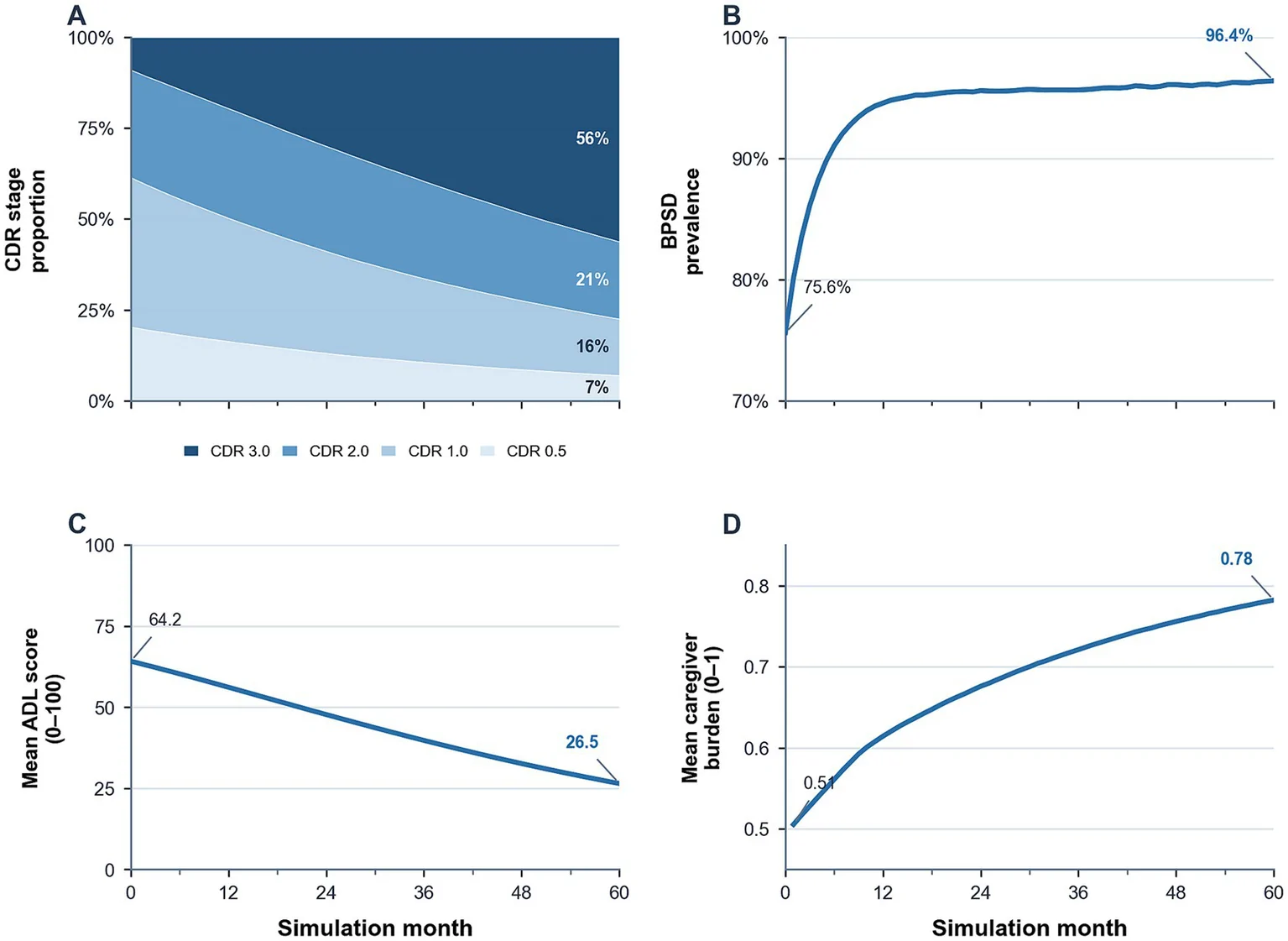
Baseline trajectories of dementia-related outcomes over 60 months. **(A)** Mean cohort proportions across Clinical Dementia Rating (CDR) stages; **(B)** Behavioral and psychological symptoms of dementia (BPSD) prevalence; **(C)** Mean activities of daily living (ADL) score; and **(D)** Mean caregiver burden. Results are based on 100 independent replications of 1,000 care units.

### 2.4 Submodels

#### 2.4.1 Disease progression

The disease-progression submodel represented cognitive impairment using four CDR stages. Stage progression was modeled as sequential monthly transitions; annual transition estimates from cohort studies were converted to monthly probabilities using $p_{\text{month}}=1-{\left(1-p_{\text{year}}\right)}^{1/12}$ (Supplementary Table S1). ADL declined at a CDR-dependent exponential rate, with more advanced cognitive impairment associated with faster decline. BPSD emergence was represented as a CDR-modified model-specific natural-history transition parameter (bpsd_monthly_emergence_rate = 0.18); completed non-pharmacological services could reduce BPSD severity and slow selected disease-progression processes. Intervention effects were time-limited, with linear decay after a specified duration of continuous use (Supplementary Table S1).

#### 2.4.2 Caregiver decisions

Caregiver decisions were generated by a rule-based engine. Help-seeking propensity was calculated by aggregating pre-specified log-odds contributions from need, prior experience, trust, economic circumstances, caregiving burden, and contextual factors, and transforming the result with a logistic function. The destination of help-seeking was selected from weighted alternatives, and intentions to use individual services were evaluated separately. The rule engine generated intentions; actual service delivery depended on the subsequent care-service process. The 10 caregiver rules and their evidence sources are documented in the Supplementary methods S4.

#### 2.4.3 Care services

The care-services submodel converted intentions into service use through sequential filters for service visibility, economic accessibility, care-seeking habits, and need matching. The service catalog included cognitive assessment, medication management, BPSD consultation, non-pharmacological intervention, caregiver support, day care, and comprehensive assessment. The financing environment incorporated insurance-specific reimbursement and CDR-proxy long-term care insurance eligibility to represent differential economic accessibility (Supplementary methods S5).

#### 2.4.4 Referral network

Referral was initiated following GP assessment under pre-specified clinical and caregiving conditions. The referral network processed episodes of upward referral, specialist acceptance, management, feedback transmission, and return to community. Upward arrival at the specialist end made the episode eligible for specialist assessment and subsequent management. A closed-loop referral required subsequent specialist management, feedback to primary care, and referral back to the community; the baseline upward-arrival probability was 0.40 (Supplementary Table S1).

#### 2.4.5 Institutional capacity

Institutionalization was represented as a care-setting transition rather than a mortality event. Admission required sustained high caregiver burden (burden ≥ 0.625 for ≥ 6 consecutive months) together with a triggering event (hospitalization or BPSD) and an institutional decision. The conditional admission probability was modified by cognitive, comorbidity, and functional factors. An economic filter represented the affordability barrier of institutional monthly costs (Supplementary methods S5). After institutionalization, a care unit no longer generated family help-seeking, community-service use, caregiver-burden, or new referral-application outcomes; disease progression, hospitalization, and system-level counts continued to be recorded.

#### 2.4.6 Death and competing risks

Death was outside the model boundary; deaths among people living with dementia arise through heterogeneous pathways (falls, infections, accidental injuries) that are distinct from the cognitive-impairment-related progression and community-care mechanisms represented here. No death state, competing-risk process, or mortality-adjusted exit was simulated.

### 2.5 Initialization

The model was initialized with a synthetic cohort of 1,000 care units. Patient and caregiver characteristics were generated from the specified empirical distributions and modeling assumptions (Supplementary methods S2).

CDR stage proportions at initialization were based on the available community-dwelling dementia evidence from Taiwan, China, reported by Huang et al. (23) ( n=276 ): 0.21, 0.40, 0.30, and 0.09 for CDR stages 0.5, 1.0, 2.0, and 3.0, respectively. BPSD prevalence was initialized before the first natural-history update. CDR-specific BPSD probabilities, derived from multi-source estimates, were aligned to the pre-specified 75.7% Chinese meta-analytic prevalence anchor (24) using a common logit-intercept adjustment ( δ=0.069 ). This procedure retained the CDR gradient while producing an initialized cohort prevalence of 75.6%; the residual 0.1-percentage-point difference reflects sampling from the synthetic cohort. The adjustment was used to align the initial cohort state (Supplementary methods S2).

The natural-history parameter governing BPSD emergence was specified independently of initialization. It represented a model-specific CDR-modified monthly transition parameter rather than a directly observed incidence rate. BPSD prevalence was recorded at month 0 before the first natural-history update and subsequently at months 1–60 after the monthly update.

### 2.6 Baseline scenario and evaluation design

The baseline scenario disabled all configuration switches and represented the modeled baseline community-care configuration.

The formal baseline comprised 100 independent simulation replications using the same 1,000-unit input cohort and a fixed set of random seeds. To support descriptive trajectory visualization, stored monthly trajectories from the frozen baseline outputs were used for BPSD and caregiver burden. CDR-stage and ADL trajectories were obtained from independent replays using the same frozen baseline code, configuration, input cohort, seed range, and 10-worker execution setting. These replays were used to display monthly trajectories; the formal endpoint summaries in Table 2 were retained from the original frozen baseline outputs, except for the closed-loop referral outcome. Implementation checks assessed the CDR-stratified BPSD gradient, month-0 recording, institutionalization exit logic, and outcome denominators. Numerical adequacy was assessed through scale diagnostics across cohorts of 100, 250, 500, and 1,000 care units (20 replications per cohort) and through cumulative replication diagnostics at *R* = 10, 25, 50, 75, and 100 for the 1,000-unit cohort (Supplementary Figure S1).

**Table 2:** Formal baseline configuration outcomes from 100 independent replications of 1,000 care units.

Outcome	Mean ± seed-level SD	MC 95% CI	Denominator
BPSD prevalence at month 0	0.756 ± 0.000	0.756–0.756	Initial cohort (initialized)
BPSD prevalence at month 60	0.964 ± 0.006	0.963–0.965	Initial patient cohort
ADL score at month 60	26.5 ± 0.4	26.4–26.6	Initial patient cohort
Cumulative hospitalizations over 60 months	2,374 ± 50	2,365–2,384	Initial patient cohort
Institutionalized care units at month 60	161 ± 10	159–163	Initial 1,000 care units
Closed-loop referral completion rate	1.68% ± 0.12%	1.66–1.71%	Initiated referral episodes
Mean caregiver burden at month 60	0.782 ± 0.004	0.781–0.783	Active community care units

MC confidence intervals describe stochastic simulation uncertainty across independent replications only.

A local one-way sensitivity analysis varied the BPSD natural-history transition parameter across five pre-specified values (0.14, 0.16, 0.18, 0.20, and 0.22). Each setting was evaluated using 1,000 care units and 100 replications; the 0.18 setting was the formal baseline. This analysis was designed to characterize the consequences of uncertainty in one influential natural-history input, rather than to identify a unique parameter value or provide a global uncertainty analysis (25) (Supplementary methods S6).

Model credibility was assessed through structural documentation, implementation checks, initialization calibration, convergence diagnostics, and pattern-oriented comparison with selected care-system observations (16, 26). Limited external longitudinal pattern-oriented comparisons are reported in the Supplementary Results S2.

### 2.7 Outcome definitions

Six primary outcomes were reported:

BPSD prevalence: proportion of patients with BPSD at month 0 (initialized) and month 60 (post-update). Denominator: initial patient cohort.

ADL score: mean ADL at month 60. Denominator: initial patient cohort (including institutionalized patients).

Cumulative hospitalizations: total hospitalization events over 60 months. Denominator: initial patient cohort; includes post-institutionalization events.

Institutionalized care units: count at month 60. Denominator: initial 1,000 care units. Represents prevalent state membership, not cumulative first admission.

Closed-loop referral completion rate: proportion of initiated referral episodes that completed upward arrival at the specialist end, specialist management, feedback to primary care, and referral back to the community during the 60-month simulation.

Mean caregiver burden: mean burden score (0–1) at month 60. Denominator: active community care units.

Monte Carlo (MC) 95% confidence intervals describe stochastic variation across 100 independent replications.

## 3 Results

### 3.1 Initialization calibration

The initialized synthetic cohort had a BPSD prevalence of 75.6% (MC 95% CI, 75.6–75.6%), compared with the prespecified Chinese meta-analytic anchor of 75.7%. The 0.1-percentage-point difference arose from sampling the 1,000-unit synthetic cohort after the CDR-stratified common logit-intercept adjustment ( δ=0.069 ).

### 3.2 Convergence diagnostics

Scale diagnostics across cohorts of 100, 250, 500, and 1,000 care units indicated that macro-level outcomes were stable at cohort sizes of 500 or more (Supplementary Figure S1a).

Cumulative replication diagnostics for the 1,000-unit cohort showed that all prespecified primary outcomes had relative Monte Carlo precision below 3% at 50 replications and below 1.5% at 100 replications. At R=100 , relative precision was 0.12% for month-60 BPSD, 0.32% for ADL, 0.40% for cumulative hospitalizations, 1.42% for institutionalization, for the original frozen endpoint outcomes (Supplementary Figure S1b). The closed-loop referral outcome was separately obtained from the independently replayed referral-process analysis.

### 3.3 Baseline configuration trajectory

Figure 2 summarizes trajectories in CDR stage distribution, BPSD prevalence, mean ADL, and mean caregiver burden under the baseline scenario. The cohort shifted toward more severe CDR stages: the mean proportion at CDR 3.0 increased from 9.0% at initialization to 56.3% at month 60, while the corresponding proportions at CDR 0.5, 1.0, and 2.0 at month 60 were 6.9%, 15.6%, and 21.2%, respectively. BPSD prevalence increased from 75.6% at initialization to 96.4% at month 60 (MC 95% CI, 96.3–96.5%). The BPSD trajectory reached 80.1% at month 1, 94.6% at month 12, and 95.6% at month 24 before approaching a later plateau.

Mean ADL declined from 64.2 at month 0 to 26.5 at month 60 (MC 95% CI, 26.4–26.6). Mean caregiver burden increased from 0.505 at month 1 to 0.782 at month 60 (0.781–0.783). At month 60, cumulative hospitalizations over 60 months were 2,374 (2,365–2,384), and a mean of 161 care units were in institutional care (159–163; Tables 2, 3). In the referral-process analysis setting, a mean of 363 closed-loop referral episodes occurred over 60 months (MC 95% CI, 358–368), equating to a closed-loop referral completion rate of 1.68% across all initiated referral episodes (1.66–1.71%).

**Table 3:** Baseline cohort characteristics at initialization (*N* = 1,000 care units).

Characteristic	Value
Patient	
Age, mean (SD)	76.6 (9.1)
Female, *n* (%)	589 (58.9)
CDR 0.5, *n* (%)	203 (20.3)
CDR 1.0, *n* (%)	411 (41.1)
CDR 2.0, *n* (%)	296 (29.6)
CDR 3.0, *n* (%)	90 (9.0)
ADL score, mean (SD)	64.2 (21.9)
BPSD present, *n* (%)	756 (75.6)
BPSD severity (among present), mean	0.60
Comorbidity count, mean	1.4
Primary caregiver	
Age, mean (SD)	60.8 (13.0)
Spouse, *n* (%)	392 (39.2)
Adult child, *n* (%)	501 (50.1)
Other, *n* (%)	107 (10.7)
Weekly care hours, mean (SD)	60.8 (28.8)
Co-caregiver present, *n* (%)	400 (40.0)
Paid carer present, *n* (%)	121 (12.1)
Monthly income > 7,000 RMB, *n* (%)	466 (46.6)
Family	
Urban residence, *n* (%)	687 (68.7)
Employee insurance, *n* (%)	812 (81.2)
Monthly ability to pay, mean (RMB)	2,428

CDR, Clinical Dementia Rating; ADL, activities of daily living (0–100); BPSD, behavioral and psychological symptoms of dementia. CDR proportions based on Huang et al. (23); BPSD prevalence reflects the initialized cohort after the common logit-intercept adjustment (delta = 0.069) aligning to the 75.7% Chinese meta-analytic anchor (24).

### 3.4 Local one-way sensitivity analysis

Varying the BPSD natural-history transition parameter from 0.14 to 0.22 yielded BPSD prevalences of 92.8–95.8% at month 12, 94.1–96.4% at month 24, and 95.5–96.9% at month 60 (Figure 3). Terminal ADL, cumulative hospitalizations, and the number of institutionalized care units changed little across this range; extended outcomes are reported in Supplementary Results S3.

**Figure 3:**
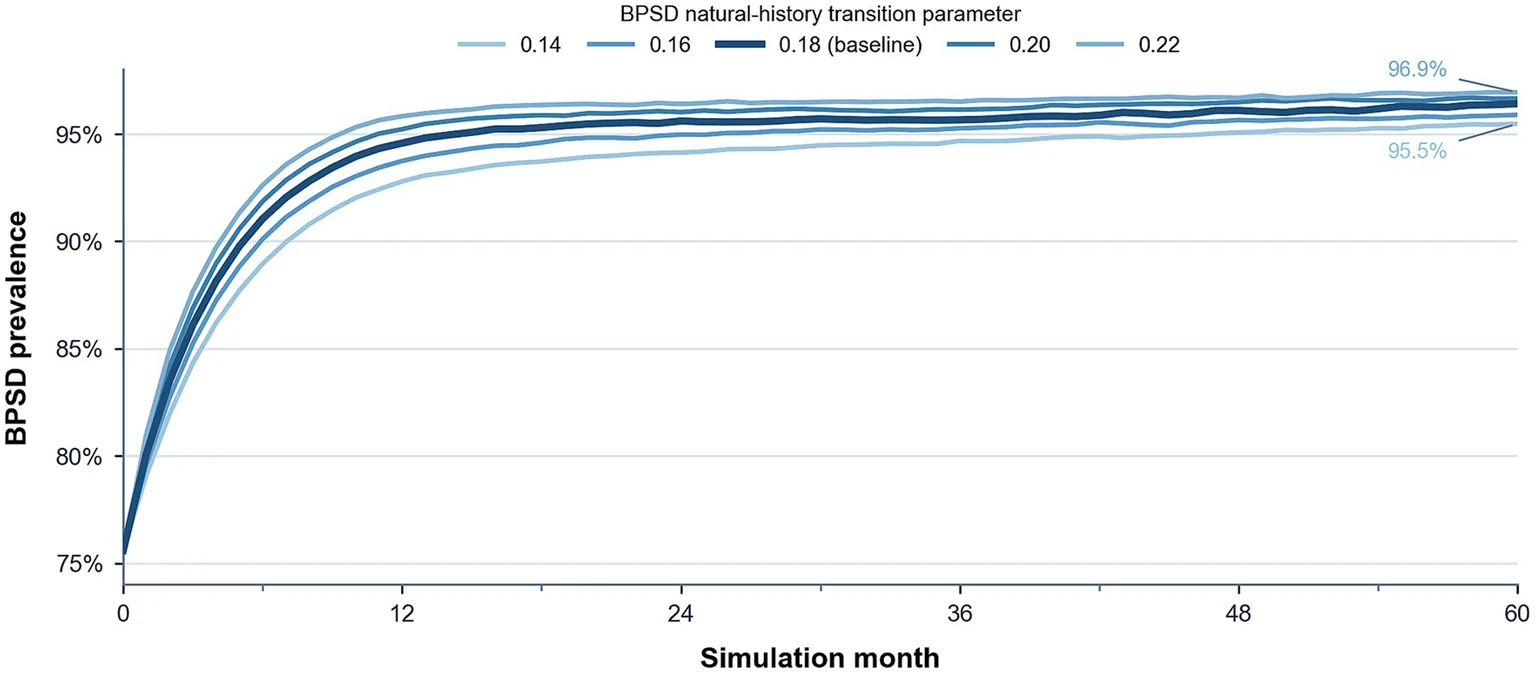
BPSD prevalence trajectories under five values of the BPSD natural-history transition parameter (0.14, 0.16, 0.18, 0.20, 0.22). Each line depicts the mean across 100 independent replications of 1,000 care units. The 0.18 setting (darker line) is the formal baseline. BPSD prevalence showed a detectable but limited response across the tested range (month-60 range: 1.5 percentage points).

### 3.5 Descriptive terminal-burden association analysis

For each care unit, terminal patient and utilization outcomes were averaged across 100 replications. Care units were ranked by their mean last observed caregiver-burden score, and the highest 5% (50/1,000) were compared with the remaining 95%. Because caregiver burden no longer entered the active community-care risk set after institutional admission, this ranking variable is not a month-60 caregiver-burden value for care units admitted before month 60.

The high-burden tail (last observed burden 0.977–0.996) had higher mean final CDR (2.80 vs. 2.27), lower final ADL (8.3 vs. 27.5), greater BPSD severity (0.76 vs. 0.59), more cumulative hospitalizations (3.06 vs. 2.34), higher initial weekly care hours (79.7 vs. 59.8), and a higher proportion in institutional care at month 60 (31.4% vs. 15.3%). Co-caregivers (0.0% vs. 42.1%) and paid carers (0.0% vs. 12.7%) were absent in the tail.

These findings are descriptive, model-generated associations. They do not identify temporal ordering, modifiable causal determinants, or mechanistic pathways into high caregiver burden.

## 4 Discussion

### 4.1 Baseline credibility and design rationale

Before computational experiments can vary organizational configurations in community dementia care, a baseline reference scenario must be established. This baseline serves as the reference object against which effects of organizational configurations will be measured. For such measurement to be attributable, the baseline must satisfy a specific requirement: every rule and parameter must be traceable to its evidence source, and the causal sequence must be inspectable by domain experts (12).

The credibility of this baseline rests on a sequence of structural conditions. Each rule and parameter traces to a documented empirical estimate or an explicitly identified assumption, and the ODD+D description makes these mappings auditable (21). Initialization calibration confirmed that the synthetic cohort’s starting point aligned with the prespecified BPSD prevalence anchor. Convergence diagnostics across 100 replications confirmed adequate reporting precision (maximum relative Monte Carlo error 1.42%). Local one-way sensitivity analysis then characterized the output range attributable to variation in a key natural-history input, bounding the current uncertainty characterization (25). These conditions together establish structural credibility proportionate to the model’s intended use as a scenario-experiment reference case, not as a population forecast or cost-utility estimate (16, 27).

Structural credibility requires that each modeled decision can be independently examined. LLM-based generative agents offer natural-language planning and interaction (28), but current validation frameworks focus chiefly on macro-level output patterns (29), and evidence on task performance and representation remains heterogeneous (30, 31), and the degree to which language-model agents reproduce human behavioral variation remains contested (34, 35). A recent critical review further noted that mechanism-level validation of LLM-driven generative ABMs remains limited (32). For the present evidentiary purpose, pre-specified rules preserve inspectable links between evidence sources, assumptions, and simulated decisions; this is a design choice, not a claim that rule-based ABMs are generally superior.

The behavior of the formal baseline configuration, including rising BPSD prevalence, accumulating burden, closed-loop referral completion, and transitions to institutional care, is therefore a conditional consequence of the documented rules. Future computational experiments will activate organizational configurations on this same model and compare outputs against this formal baseline configuration, thereby characterizing the conditional consequences of alternative arrangements.

### 4.2 Scope, boundaries, and next steps

The interpretation of the formal baseline configuration is bounded by several structural choices. Model outputs are conditional consequences of the documented rules, parameters, and initialized cohort, rather than direct predictions of population-level outcomes or estimates of real-world intervention effects. Death and competing risks were not represented, and institutionalization was modeled as a transition in care setting. The initialized CDR distribution was drawn from a community-dwelling dementia sample from Taiwan, China, which supports synthetic-cohort construction but does not support direct geographic generalization to mainland populations. In addition, specialist care was represented as a functional service node rather than a population of clinicians or institutions with heterogeneous practice styles and capacity constraints.

Future work should assess external validity against longitudinal empirical data, incorporate mortality and competing risks, and examine whether key assumptions remain robust across settings with different service capacity and organizational arrangements. The present model is intended to provide an inspectable comparison condition for such work. Extensions could further draw on need-driven dementia-compromised behavior perspectives to specify how unmet needs and environmental triggers shape BPSD-related help-seeking and care responses. In all cases, policy or organizational scenarios, as well as any additional mechanisms, should be introduced only when their mechanisms, parameterization, and intended comparison conditions are prospectively specified and empirically evaluated (33).

## 5 Conclusion

This study reports a transparent, rule-based ABM of community dementia care and a formally assessed baseline configuration. The model translates heterogeneous evidence into explicit rules and parameters, aligns the initialized cohort with a prespecified BPSD anchor, and documents simulation behavior through implementation checks, convergence assessment, and local sensitivity analysis. Under its documented assumptions, the formal baseline configuration generates conditional trajectories across patient, caregiver, service-use, and care-setting domains. Its contribution is a traceable, inspectable, and reconfigurable comparison condition for future, explicitly scoped scenario analyses of organizational configurations in community dementia care.

## Data Availability

The original contributions presented in the study are included in the article/Supplementary material, further inquiries can be directed to the corresponding authors.
