# Supplementary material for: Evidence-based construction and validation of an agent-based model of community integrated care for older adults living with dementia
Source: Front Public Health. 2026 Sep 23;14:1913457. doi: 10.3389/fpubh.2026.1913457 (PMC13642730; doi:10.3389/fpubh.2026.1913457)
Supplement: Supplementary file 1 [file Data_sheet_1.pdf]

Evidence-Based Construction and Validation of an Agent-Based Model of Community Integrated Care for Older Adults Living with Dementia -- Supplementary Material

Supplementary Methods S1. Evidence grading system and registry summary

Each parameter and rule in the evidence registry was graded on two independent dimensions.

Dimension A - Population provenance:

Level	Definition	Example
L1	Beijing/local primary data from Q1-Q5	Q1 interview-derived help-seeking thresholds
L2	Other Chinese empirical data	Huang 2017 Taiwan CDR distribution; Ding 2025 Chinese BPSD meta-analysis
L3	International empirical data from comparable systems	Tariot 2024 NACC CDR transitions; Shepherd 2019 hospitalization meta-analysis
L4	Qualitative/directional evidence	Directional evidence chains from multi-study synthesis

Dimension B - Evidence strength:

Level	Definition
A	Systematic review/meta-analysis or large prospective cohort (n > 1,000)
B	Single prospective cohort or large cross-sectional survey (n > 200)
C	Small cohort, cross-sectional study, or qualitative study
D	Directional evidence or model-specified natural-history input; no directly estimated quantitative effect size available

Evidence registry summary

The evidence registry contains 150 records: 76 behavioral/interaction rules, 24 initialization parameters, 20 environment parameters, 11 constraints, 9 agent parameters, and 4 configuration parameters. The 21 disease progression parameters listed in Table S1, the 10 caregiver rules in Table S2, and the 11 burden estimator parameters in Table S3 constitute the core parameter set with full evidence documentation. The complete evidence registry, including all 150 records with full source citations, population provenance, evidence-strength assessments, and linked model mechanisms, is maintained as a curated spreadsheet and will be deposited alongside the model code in a public repository upon publication.

Supplementary Methods S2. ODD+D protocol declaration

The model follows the ODD+D protocol (20,27). This supplementary section documents the design concepts.

S2.1 Purpose, entities, state variables, and scales

See main text Sections 2.1 and 2.2.

S2.2 Process overview and scheduling

See main text Section 2.3 and Figure 1.

S2.3 Design concepts

Basic principles. The model is fully rule-based. All decision processes are specified as pre-specified rules, thresholds, or probabilistic functions parameterized through log-odds weights.

Emergence. System-level patterns emerge from local interactions among care units, providers, and institutions. Key emergent outcomes include BPSD prevalence trajectories (from individual BPSD emergence/severity processes), service uptake rates (from individual help-seeking intentions filtered through visibility, economic, habit, and completion gates), unmet-need stratification by barrier type (economic, visibility, habit), and institutionalization counts (from individual burden accumulation and trigger events).

Adaptation. Caregivers do not perform explicit learning or optimization. Three implicit adaptation mechanisms operate: (1) prior-use inertia, services previously used receive a log-odds bonus (+0.30) in subsequent help-seeking (R05); (2) trust accumulation, coordinator contacts below a threshold (3) reduce help-seeking propensity (-0.10), while contacts at or above the threshold increase it (+0.30) (R13); (3) fatigue accumulation, consecutive months of high burden add a fatigue term (capped at 0.25) to the burden estimate. GP competency can grow monthly when the corresponding configuration is enabled.

Objectives. Caregivers seek to address care needs through help-seeking and service use. GPs aim to assess, manage, and refer as indicated by clinical thresholds. Specialists aim to diagnose, manage, and return patients to community care. None of these objectives are explicitly optimized; they are encoded as rule-based decision processes.

Learning. No explicit learning occurs. GP competency growth (when enabled) is a parameterized monthly increment, not a learning algorithm. Caregiver trust accumulation is a count-based state variable, not a learned policy.

Sensing. Caregivers perceive patient status (CDR, BPSD, ADL) from the blackboard, service visibility and affordability from the care-service system, coordinator contacts, prior service-use history, and configuration flags (if enabled). GPs perceive caregiver help-seeking decisions, burden levels, and patient status. Specialists perceive referral requests.

Interaction. Agents interact indirectly through a blackboard architecture. Key interaction chains: caregiver to GP (help-seeking signals), GP to specialist (referral), specialist to GP (feedback), GP to caregiver (assessment unlocks service visibility), caregiver to disease progression (service use affects BPSD/ADL), disease progression to caregiver (patient status updates), disease progression to institution (institutionalization applicants).

Stochasticity. Random processes include CDR stage transitions, BPSD emergence and symptom selection, hospitalization, institutionalization admission, help-seeking decisions, help-seeking target selection, service-use decisions, burden noise (Gaussian), habit gating, and referral completion. All RNG streams are seeded deterministically from a base seed, ensuring reproducibility.

Observation. Per-epoch, the model records BPSD prevalence, ADL mean, hospitalization events, institutionalization status, service uptake, unmet-need events by barrier type, economic indicators (gross cost, payer payment, household out-of-pocket, OOP-to-ability ratio), GP action frequencies, referral completion, and caregiver burden. End-of-run summaries include per-seed terminal outcomes and per-care-unit trajectories.

Collectives. No explicit collective structures (groups, networks) are modeled. Care units are the primary unit of analysis. GPs are associated with institutions but do not form explicit networks.

Heterogeneity. Caregivers are heterogeneous in demographics, socioeconomic status, caregiving intensity, and prior service use. GPs are heterogeneous across three competence dimensions (assessment, medication management, and BPSD management); competence values are assigned at the individual level and, when the learning-organization configuration is enabled, increase at institution-stratified rates. Care coordinators are available only when the corresponding configuration is enabled and have limited contextual heterogeneity. A fixed, deterministically sampled fraction of care units is assigned a social-worker-type coordinator whose support competence is set to at least 0.50; support competence for other coordinators depends on institutional competence and can change under the learning-organization configuration. Coordinator background, experience, workload, and practice style are not represented. Hospital specialists are represented as network-level functional nodes; individual specialist competence, clinical style, and capacity heterogeneity are intentionally outside the model boundary.

State transitions and outcome denominators. Institutionalization is a care-setting transition, not death, censoring, or a competing event. Institutionalized units no longer contribute family help-seeking, community-service use, caregiver-burden, or new referral-application outcomes, but disease progression, hospitalization, and system-level counts continue. BPSD prevalence, ADL, and cumulative hospitalizations use the initial patient cohort as denominator; institutionalization is the month-60 state count in the initial care-unit cohort; referral completion and caregiver burden use active community care units.

S2.4 Initialization

See main text Section 2.5. The CDR distribution was drawn from Huang et al. (29). Before the first natural-history update, CDR-specific BPSD probabilities derived from multi-source estimates were shifted by a common logit intercept (delta = 0.069) to align the initialized synthetic cohort with the prespecified 75.7% Chinese meta-analytic prevalence anchor (30); the realized month-0 prevalence was 75.6% because of finite-cohort sampling. The BPSD monthly emergence parameter (0.18) is analytically distinct from this initialization procedure: it is a model-specific, CDR-modified monthly natural-history transition input, not a directly observed incidence rate and not a parameter fitted to the 75.7% anchor. Ten alternative initial CDR compositions required delta values from -0.20 to +0.45 to reach the same cross-sectional anchor. This exploration demonstrates that different initial compositions can be aligned through different intercept adjustments; it is an internal initialization robustness check, not joint parameter calibration, global uncertainty analysis, or structural-uncertainty quantification.

S2.5 Input data

See main text Section 2.1 and Table 1.

Supplementary Methods S3. Disease progression parameters

Table S1. Disease progression parameters.

Parameter	Default	Evidence	Grade	SA range
CDR 0.5 to 1.0 monthly transition	0.0185	Tariot 2024 NACC n=28,220	A	-
CDR 1.0 to 2.0 monthly transition	0.0254	Tariot 2024 NACC	A	-
CDR 2.0 to 3.0 monthly transition	0.0306	Tariot 2024 NACC	A	-
ADL monthly decline rate	0.002	Yi 2020 Rasch calibration	B	-
ADL decline CDR coefficient	2.0	Yi 2020	B	-
BPSD monthly emergence rate	0.18	Model-specific CDR-modified natural-history input; analytically distinct from the Ding 2025 75.7% initialization anchor	D	[0.14, 0.22]
Non-pharma CDR slowdown	0.337	Desai 2024 CST d=0.45	C	[0.20, 0.50]
Non-pharma ADL slowdown	0.08	Desai 2024	C	[0.00, 0.20]
Non-pharma BPSD relief	0.225	Desai 2024 BPSD d=-0.36	C	[0.12, 0.35]
Medication CDR slowdown	0.083	Multi-source	B	[0.04, 0.15]
Intervention decay onset month	6	Xiao 2024	B	[4, 8]
Intervention decay end month	9	Xiao 2024	B	[8, 12]
Hospitalization rate CDR 0.5	0.013	Shepherd 2019 + Chinese registry	A	-
Hospitalization rate CDR 1.0	0.025	Shepherd 2019	A	[0.015, 0.040]
Hospitalization rate CDR 2.0	0.042	Shepherd 2019	A	-
Hospitalization rate CDR 3.0	0.059	Shepherd 2019	A	-
Hospitalization absence multiplier	2.0	Amjad/Wolff 2021 strain aHR=1.21	C	[1.5, 3.0]
Institutionalization trigger prob	0.10	Bruck 2025 + Yaffe 2002	A+B	[0.05, 0.20]
Burden high threshold	0.625	Yaffe 2002 ZBI Q4 >= 20 (HR=1.73)	B	[0.50, 0.75]
Collapse streak threshold	6 months	Model assumption	D	[3, 12]

Parameter	Default	Evidence	Grade	SA range
Reverse exacerbation factor	1.5	Directional evidence (qualitative synthesis)	D	[1.0, 2.0]

SA = sensitivity analysis. Only bpsd_monthly_emergence_rate was evaluated in the completed local one-way SA. Other SA ranges represent pre-specified perturbation bounds for future analyses. The 0.18 BPSD transition input was not fitted to the 75.7% cross-sectional anchor; that anchor was used only for the month-0 common logit-intercept initialization adjustment (delta = 0.069). Grade E, where used in the evidence registry, denotes a parameter aligned to an empirical anchor through a documented model-alignment procedure but not directly estimated from a single source.

Supplementary Methods S4. Caregiver rule engine

S4.1 Rule list

Ten rules (R01-R17, non-sequential numbering) generate help-seeking propensity, service-use intentions, and burden adjustments.

Table S2. Caregiver rules and evidence sources.

Rule	Description	Evidence source	Contribution type
R01	Baseline help-seeking propensity (role-stratified: spouse approximately 0.15, adult child approximately 0.30)	Q1	help_seeking log-odds
R02	Private network dependence (formal help-seeking penalty -0.50; spouse x1.2)	Q1	help_seeking log-odds
R03	Safety risk elevation (agitation/wandering or ADL < 40 leads to +0.50)	Q1	help_seeking log-odds
R04	Rural attenuation (multiplier 0.50)	Q4	help_seeking log-odds
R05	Prior-use inertia (each previously used service +0.30)	Q4	service log-odds
R06	Hospital preference (knowing memory clinic leads to hospital weight 2.00; not knowing leads to 0.30)	Q2	target weights
R07	Income effect (monthly income > 7,000 RMB leads to non-pharma OR=4.15; day care x0.5; caregiver support x0.3)	Q3	service log-odds
R12	Coordinator effect (configuration enabled + contact leads to +0.40)	Q4	help_seeking log-odds
R13	Trust accumulation (contacts < 3: -0.10; contacts >= 3: +0.30)	Q4, Q5	help_seeking log-odds
R17	Spouse collapse threshold adjustment (spouse burden_delta = -0.02 x streak_diff)	Q1	burden delta

S4.2 Aggregation

Help-seeking propensity is calculated by aggregating log-odds contributions from applicable rules and transforming the result with a logistic function. The destination of help-seeking is selected from weighted alternatives (hospital, GP, community, family). Intentions to use individual services are evaluated separately through per-service log-odds. The rule engine generates intentions; actual service delivery depends on the subsequent care-service process (Supplementary Methods S5).

Illustration of evidence-to-rule translation. Directional or qualitative evidence was used to specify the applicable decision condition, direction of contribution, and prespecified plausible range; it was not treated as a directly estimated causal effect size. For example, the applicable baseline contribution for a primary caregiver is R01_base_logodds_primary = -1.70; when coordinator contact is applicable, R12 adds 0.40; and R13 contributes -0.10 before three contacts and +0.30 at or above three contacts. The applicable contributions are summed to a total log-odds value L, and help-seeking probability is calculated as $1/(1 + e^{-L})$. The resulting intention then remains subject to the service visibility, affordability, habit, and completion filters.

S4.3 Burden estimator

Caregiver burden is estimated monthly using the following formula:

```
burden = base
       + cdr_direct_weight * patient_cdr
       + bpsd_direct_weight * patient_bpsd_severity
       + intensity_weight * min(1.0, weekly_care_hours / 120.0)
       + min(fatigue_cap, high_burden_streak * fatigue_rate)
       + role_bonus (if primary caregiver)
       - support_co_caregiver (if co-caregiver present)
       - support_hired (if paid carer present)
       + gaussian_noise(0, noise_sd)
```

The result is clamped to [0, 1]. Weekly care hours are derived from baseline care hours multiplied by an exponential function of ADL loss (Reed et al. 2016: each 1-point ADL decline leads to 4.1% increase in care hours).

Table S3. Burden estimator parameters.

Parameter	Default value	Evidence source	SA range
burden_base	0.30	Calibration baseline	-
burden_cdr_direct_weight	0.045	Retained from legacy formula	-
burden_bpsd_direct_weight	0.150	Shim 2016 / Takechi 2024 (standardized beta approximately 0.37-0.41); model-calibrated	[0.100, 0.200]
burden_intensity_weight	0.20	Care-hours weight	-
burden_fatigue_rate	0.03	Monthly high-burden accumulation	-
burden_fatigue_cap	0.25	Fatigue accumulation ceiling	-
burden_role_bonus	0.05	Spousal additional burden	-
burden_support_co_caregiver	0.10	Co-caregiver relief	-
burden_support_hired	0.15	Paid carer relief	-
burden_noise_sd	0.05	Individual variation SD	[0.02, 0.10]
care_hours_adl_log_rate	0.03134	Reed 2016 (ADCS-ADL 1-point decline leads to 4.1% increase)	[0.02759, 0.03508]

S4.4 Behavioral economic layer

A behavioral economic gating layer operates in the intention-generation phase, independent of the burden estimator. When the OOP-to-ability ratio for a service exceeds a threshold, a penalty is applied to the service-use log-odds:

```
penalty = econ_beta * min(ratio, econ_ratio_cap)
total_logodds -= penalty
```

Parameters: econ_intent_relative_burden_beta = 1.0 (SA [0.5, 1.0, 2.0]); econ_intent_relative_burden_cap = 3.0. A hard economic gate (OOP > effective ability to pay) still operates at the service execution stage.

Supplementary Methods S5. Service economic structure

S5.1 Service catalogue

Table S4. Service catalogue and economic parameters.

Service	Type	Monthly cost (RMB)	Reimbursable	Reimbursement rate (employee/resident)	LTCI-eligible
Cognitive assessment	Diagnosis	64	Yes	70% / 50%	No
Medication management	Treatment	500	Yes	70% / 50%	No
BPSD consultation	Treatment	400	No	0%	No
Non-pharmacological intervention	Rehabilitation	1,500	Yes	70% / 50%	No
Caregiver support	Support	600	No	0%	No
Day care	Community	3,600	No	0%	Yes (CDR >= 2.0)
Comprehensive assessment	Professional assessment	170	Yes	70% / 50%	No

S5.2 LTCI subsidy

Long-term care insurance (LTCI) eligibility is based on CDR proxy: - Eligibility threshold: CDR >= 2.0 - Monthly subsidy: CDR 2.0 leads to 1,000 RMB; CDR 3.0 leads to 2,000 RMB - Directed services: subsidy applies only to day care

S5.3 Four-layer service filtering

Service delivery follows a sequential four-layer filter:

1. **Information visibility:** Services are visible if (a) known to the care unit, (b) unlocked by GP assessment, (c) broadcast by information system (when configuration enabled), or (d) unlocked by completed referral.
2. **Economic accessibility:** Out-of-pocket cost is calculated as gross cost minus insurance payment minus LTCI subsidy. If OOP exceeds effective ability to pay, the service is blocked (economic barrier).
3. **Care-seeking habit:** A probabilistic gate based on residence type (urban: 0.610; rural: 0.350), adjusted by prior-use bonus (+0.20) and proactive outreach bonus (+0.15, if enabled).
4. **Completion probability:** Base completion probability (0.477), adjusted by coordination (0.78, if coordinator configuration enabled and coordinator contacted).

S5.4 Institutionalization economic filter

Institutional admission requires that monthly resources (household budget + LTCI subsidy) meet or exceed the institution monthly cost (12,000 RMB). If affordability is not met, the admission probability is reduced to 10% (hardship probability, representing asset liquidation or borrowing).

Supplementary Methods S6. Sensitivity analysis parameter bounds

Only the BPSD monthly emergence input was evaluated in the completed local one-way sensitivity analysis (five values, 0.14-0.22; 1,000 care units and 100 replications per value). The remaining ranges in Table S5 are prespecified parameter-governance bounds for future analyses; they do not represent completed joint sampling, global sensitivity analysis, uncertainty propagation, alternative-structure testing, or configuration-effect uncertainty analysis.

Table S5. Pre-specified sensitivity analysis parameter bounds (67 parameters in 7 groups).

Group A: High priority (8 parameters)

Parameter	Description	Default	Low	High
reverse_exacerbation_factor	Burden-to-BPSD amplification factor when burden is sustained high	1.5	1.0	2.0
nonpharma_adl_slowdown	ADL decline reduction from non-pharmacological intervention	0.08	0.00	0.20
collapse_streak_threshold	Months of sustained high burden for institutionalization trigger	6	3	12
hospitalization_monthly_rate_by_cdr.1.0	Monthly hospitalization probability for CDR 1.0	0.025	0.015	0.040
hospitalization_absence_multiplier	Hospitalization probability multiplier during caregiver absence	2.0	1.5	3.0
burden_high_threshold	Caregiver burden threshold for institutionalization eligibility	0.625	0.50	0.75
decay_onset_month	Month when intervention effect begins to decay	6	4	8
decay_end_month	Month when intervention effect fully decays	9	8	12

Group B: Medium priority (14 parameters)

Parameter	Description	Default	Low	High
caregiver_attention_prob_base	Base probability of caregiver attention to patient needs	0.20	0.10	0.35
caregiver_attention_prob_s1	Caregiver attention probability with S1 enabled	0.60	0.40	0.80
referral_prob_no_agreement	Referral probability without	0.40	0.25	0.60

Parameter	Description	Default	Low	High
	formal agreement			
referral_prob_with_agreement	Referral probability with formal agreement	0.75	0.60	0.90
institution_high_capacity_fraction	Fraction of institutions with high capacity	0.127	0.10	0.20
institution_high_competency_gain	Monthly competency gain for high-capacity institutions	0.02	0.015	0.025
institution_low_competency_gain	Monthly competency gain for low-capacity institutions	0.01	0.005	0.015
base_referral_completion_prob	Base probability of referral completion	0.40	0.25	0.55
risk_crisis_threshold	Risk score threshold for crisis-level proactive outreach	0.75	0.65	0.85
risk_high_threshold	Risk score threshold for high-level proactive outreach	0.55	0.45	0.65
risk_medium_threshold	Risk score threshold for medium-level proactive outreach	0.35	0.25	0.45
admission_acceptance_prob	Probability of institutional admission acceptance	0.80	0.60	0.95
s1_outreach_propensity_bonus	Proactive outreach propensity bonus (S1)	0.15	0.05	0.25
s1_outreach_interval_months	Interval between proactive outreach attempts (S1)	3	1	6

Group C: Low priority (14 parameters)

Parameter	Description	Default	Low	High
nonpharma_cdr_slowdown	CDR progression reduction from non-pharmacological intervention	0.337	0.20	0.50
nonpharma_bpsd_relief	BPSD severity reduction from non-pharmacological intervention	0.225	0.12	0.35
medication_cdr_slowdown	CDR progression reduction from medication management	0.083	0.04	0.15
institutionalization_trigger_prob	Base probability of institutionalization trigger	0.10	0.05	0.20
care_seeking_habit_prob.rural	Care-seeking habit probability for rural residents	0.35	0.20	0.50
manage_and_refer_back_prob_no_agreement	Probability of manage-	0.30	0.15	0.45

Parameter	Description	Default	Low	High
	and-refer-back without agreement			
manage_and_refer_back_prob_with_agreement	Probability of manage-and-refer-back with agreement	0.70	0.50	0.90
burden_referral_threshold	Burden threshold for referral initiation	0.80	0.70	0.90
new_bpsd_referral_window_months	Window for BPSD-triggered referral	3	1	6
bpsd_severity_referral_threshold	BPSD severity threshold for referral	0.70	0.50	0.90
s4_broadcast_coverage	Information system broadcast coverage (when enabled)	0.90	0.70	1.00
specialist_diagnosis_competency_bonus	Competency bonus from specialist diagnosis	0.10	0.05	0.15
specialist_diagnosis_expiry_months	Validity period of specialist diagnosis	6	3	12
record_threshold_bonus	Record-keeping threshold bonus	0.05	0.02	0.08

Group D: Caregiver rule directional (15 parameters)

Parameter	Description	Default	Low	High
R01_base_logodds_primary	Base help-seeking log-odds for primary caregivers	-1.70	-2.10	-1.10
R01_s1_bonus	Help-seeking log-odds bonus with S1	1.00	0.50	1.50
R03_safety_bonus	Help-seeking log-odds bonus for safety risk	0.50	0.20	0.80
R04_rural_multiplier	Rural residence help-seeking multiplier	0.50	0.30	0.70
R05_prior_use_bonus	Prior service use log-odds bonus	0.30	0.10	0.50
R06_hospital_weight_aware	Hospital target weight when memory clinic known	2.00	1.00	3.00
R12_coordinator_bonus	Coordinator contact help-seeking bonus	0.40	0.20	0.60
R13_trust_threshold	Trust accumulation threshold for help-	3	2	4

Parameter	Description	Default	Low	High
	seeking bonus			
R13_post_trust_logodds	Help-seeking log-odds after trust threshold	0.30	0.10	0.50
R17_collapse_streak_diff	Spousal collapse streak difference adjustment	2	1	4
burden_noise_sd	Burden estimation Gaussian noise SD	0.05	0.02	0.10
care_hours_adl_log_rate	ADL-to-care-hours log rate	0.03134	0.02759	0.03508
burden_bpsd_direct_weight	BPSD severity weight in burden estimator	0.150	0.100	0.200
service_base_prob.non_pharma_intervention	Base probability of non-pharma intervention use	0.15	0.05	0.30
service_base_prob.day_care	Base probability of day care use	0.10	0.03	0.20

Group E: Natural-history input (1 parameter)

Parameter	Description	Default	Low	High
bpsd_monthly_emergence_rate	Model-specific CDR-modified monthly BPSD emergence input (not fitted to the 75.7% initialization anchor)	0.18	0.14	0.22

Group F: Economic accessibility (9 parameters)

Parameter	Description	Default	Low	High
ltci_subsidy_by_cdr.2	LTCI monthly subsidy for CDR 2.0	1000	500	1500
ltci_subsidy_by_cdr.3	LTCI monthly subsidy for CDR 3.0	2000	1000	3000
ltci_eligible_cdr_threshold	CDR threshold for LTCI eligibility	2.0	1.0	3.0
econ_intent_relative_burden_beta	Economic burden penalty coefficient	1.0	0.5	2.0
day_care_base_cost	Day care monthly cost	3600	2500	5000
reimbursement_rate.employee	Employee insurance reimbursement rate	0.70	0.55	0.85
reimbursement_rate.resident	Resident insurance reimbursement rate	0.50	0.35	0.60
cognitive_assessment_interval_months	Cognitive assessment interval	12	6	12
comprehensive_assessment_interval_months	Comprehensive assessment interval	6	3	12

Group G: Conduction pathways (6 parameters)

Parameter	Description	Default	Low	High
gp_medication_cdr_slowdown	CDR progression reduction from GP medication management	0.20	0.15	0.25
coordinator_bpsd_relief	BPSD severity reduction from coordinator contact	0.05	0.02	0.08
coordinator_burden_relief	Burden reduction from coordinator contact	0.05	0.02	0.08
specialist_feedback_competency_bonus	GP competency bonus from specialist feedback	0.10	0.05	0.15
s1_referral_intent_bonus	Referral intent bonus with S1	0.10	0.05	0.15
nonpharma_coordination_gate	Coordination gate for non-pharma intervention	0.50	0.40	0.60

Supplementary Tables

Table S6. Operationalization of the five integrated-care configurations (not evaluated in this manuscript).

All switches were set to false in the formal baseline. The configurations are implementation specifications derived from the study's empirical evidence base and structured expert consultation; they are not intervention-effect estimates. The strategy resolver activates the relevant switches, while the corresponding rule agents and subsystems apply the parameterized mechanisms described below.

Configuration	Targeted barrier and eligibility	Implemented mechanism and parameters	Explicit model boundary
S1. Proactive outreach	Unmet recognition and delayed help-seeking. Among community-dwelling units without reactive help-seeking in the current month, eligibility requires CDR >= 2 or BPSD; attempts occur every 3 months.	Proactive pathway is mutually exclusive with the reactive pathway. Caregiver attention probability changes from 0.20 to 0.60; s1_outreach_propensity_bonus = 0.15; R01_s1_bonus = 1.00; s1_referral_intent_bonus = 0.10.	Does not add staff, change geographic distance, or force service uptake.
S2. Bidirectional referral agreement	Fragmented referral, specialist receipt, feedback, and return to community.	Referral completion changes from 0.40 to 0.65; GP referral probability from 0.40 to 0.75; specialist acceptance from 0.70 to 0.90; feedback from 0.20 to 0.80; management and return to community from 0.30 to 0.70.	Does not represent queues, appointment delay, specialist capacity, or individual specialist practice variation.
S3. Care coordinator	Need for risk-stratified follow-up and cross-service coordination among active community units.	Risk score weights: CDR 0.25, ADL 0.20, BPSD 0.20, caregiver burden 0.25, help-seeking 0.10. Crisis, high, and medium thresholds: 0.75, 0.55, and 0.35. Coordinated-service completion changes from 0.477 to 0.78; coordinator contact provides BPSD and burden relief of 0.05 each, R12 bonus 0.40, and R13 trust threshold 3.	No individual coordinator workload, caseload, background, or practice-style distribution. Limited contextual heterogeneity is described in S2.3.
S4. Condition-specific information and shared record	Information discontinuity and limited service visibility.	Structured record and information coverage is 0.90. The system broadcasts cognitive assessment, non-pharmacological intervention, caregiver support, and day care; it adds 0.10 to GP assessment competence, lowers coordinator risk thresholds by 0.05, and provides a 0.10 GP competence bonus for 6 months after specialist diagnosis.	Does not model interoperable technical standards, data quality, privacy processes, or user interface burden.
S5. Learning organization and institutional capacity	Stagnant organizational capability.	Global institutional competence begins at 0.40 and gains 0.01 per month when enabled, capped at 0.90. GPs retain their initial three-dimensional heterogeneity and grow	Does not add personnel, specialist capacity, budget, or mandatory bed

Configuration	Targeted barrier and eligibility	Implemented mechanism and parameters	Explicit model boundary
		at 0.02/month in high-competence institutions and 0.01/month in low-competence institutions, subject to the same ceiling.	capacity. It is a parameterized capability-development mechanism, not an explicit learning algorithm.

Supplementary Results S1. Convergence diagnostics

S1.1 Scale diagnostics (R=20 per cohort size)

Outcome	N=100	N=250	N=500	N=1000
Month 60 BPSD	0.964 +/- 0.009	0.961 +/- 0.006	0.964 +/- 0.003	0.964 +/- 0.003
Final ADL	27.0 +/- 0.8	27.3 +/- 0.4	27.0 +/- 0.4	26.5 +/- 0.2
Hospitalizations	232 +/- 5	594 +/- 10	1,175 +/- 13	2,396 +/- 15
Institutionalized	17 +/- 2	40 +/- 2	83 +/- 3	161 +/- 5

S1.2 Replication diagnostics (N=1,000)

**Outcome**	**R=10**	**R=25**	**R=50**	**R=100**
Month 60 BPSD (relative precision)	0.48%	0.25%	0.18%	0.12%
Final ADL	0.96%	0.63%	0.43%	0.32%
Hospitalizations	0.65%	0.57%	0.57%	0.40%
Institutionalized	4.81%	3.06%	2.23%	1.42%
Referral completion	1.88%	1.39%	1.18%	0.87%

Supplementary Results S2. External longitudinal pattern-oriented comparisons

S2.1 Hospitalization

Simulated hospitalization intensity (0.475 events per nominal person-year) fell within the broad international range of 0.37-1.26 events per person-year reported in dementia studies. Time origin, denominators, event definitions, measurement scales, baseline severity, mortality, and competing-risk handling differ between the model and comparator studies.

S2.2 ADL decline (ALSOVA comparison)

Year	Model mean (0-100)	Model % of scale	ALSOVA ADCS-ADL (0-78)	ALSOVA % of scale
0	64.2	64.2%	64.6	82.8%
1	56.2	56.2%	58.1	74.5%
2	47.8	47.8%	51.3	65.7%
3	39.8	39.8%	46.1	59.0%
4	32.7	32.7%	43.6	55.9%
5	26.5	26.5%	38.0	48.7%

Model range-normalized annual decline: 7.54 percentage points/year. ALSOVA range-normalized annual decline: 6.83 percentage points/year. The scales, baseline disease severity, care settings, and handling of institutionalization and death differed.

Ref: Saari T, Hallikainen I, Hanninen T, Raty H, Koivisto A. Relationships between cognition and activities of daily living in Alzheimer's disease during a 5-year follow-up: ALSOVA Study. J Alzheimers Dis. 2018;64(1):269-279. doi:10.3233/JAD-171059.

Supplementary Results S3. Extended sensitivity analysis outcomes

Varying bpsd_monthly_emergence_rate from 0.14 to 0.22 (N=1,000, R=100 per level). This table provides the extended outcomes (ADL, institutionalization, hospitalizations).

Outcome	Rate=0.14	Rate=0.16	Rate=0.18 (baseline)	Rate=0.20	Rate=0.22
Month 12 BPSD	0.931	0.940	0.946	0.955	0.960
Month 24 BPSD	0.942	0.950	0.956	0.961	0.964
Month 60 BPSD	0.955	0.959	0.964	0.967	0.969
Final ADL	26.5	26.5	26.5	26.5	26.5
Institutionalized	159	158	161	158	161
Hospitalizations	2,379	2,388	2,374	2,389	2,380

BPSD showed a detectable but limited response (month-60 range: 1.5 percentage points). ADL, institutionalization, and hospitalization changed little across the tested range.

Supplementary Figure S1

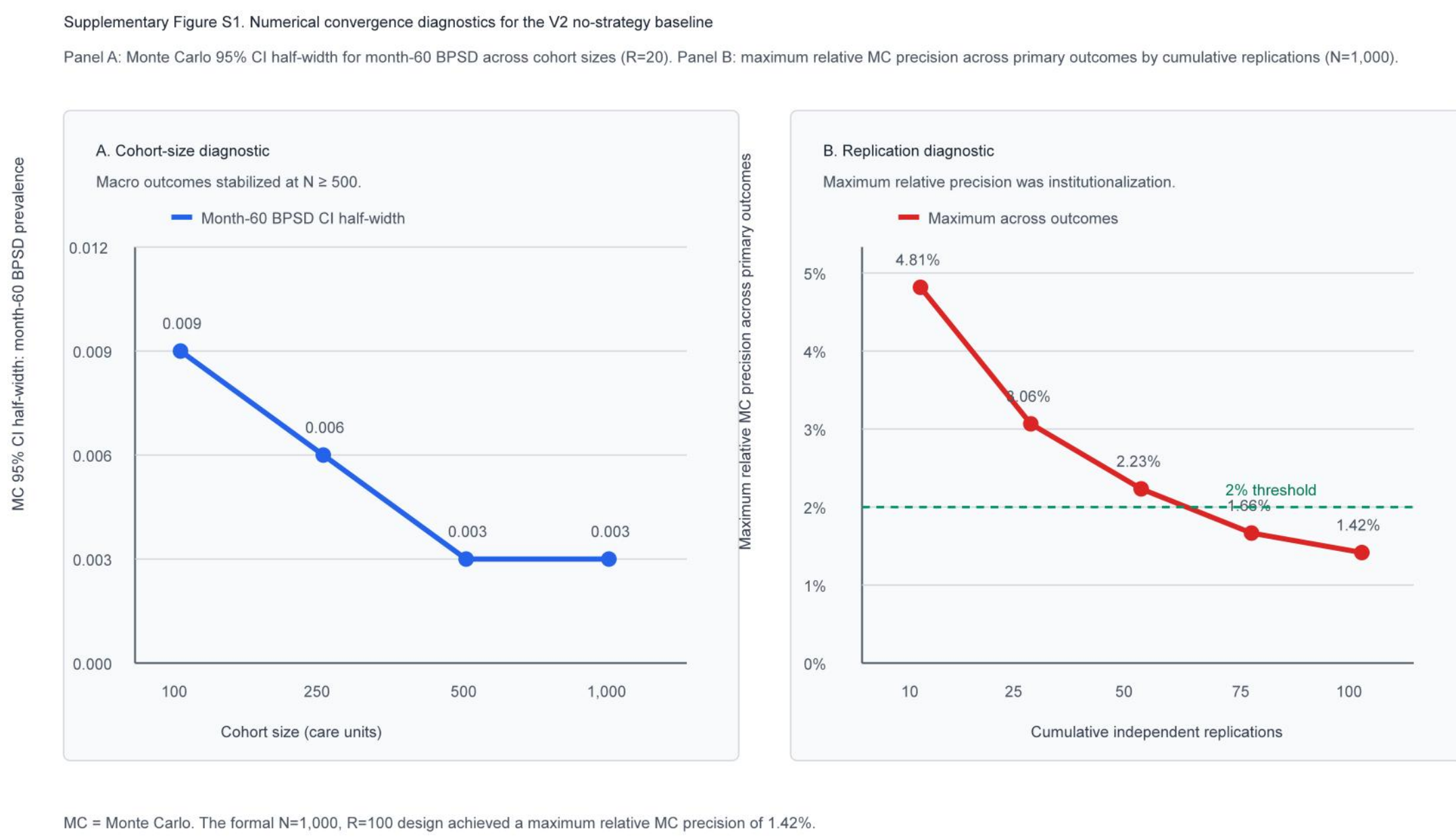

Figure S1. Numerical convergence diagnostics. (A) Scale diagnostics across cohorts of 100, 250, 500, and 1,000 care units (20 replications per cohort), showing macro-level outcome stability at N >= 500. (B) Cumulative replication diagnostics at R = 10, 25, 50, 75, and 100 for the 1,000-unit cohort, showing relative Monte Carlo precision below 3% at R = 50 and below 1.5% at R = 100 for all primary outcomes.
